# Health systems efficiency in Eastern Mediterranean Region: a data envelopment analysis

**DOI:** 10.1186/s12962-020-00217-9

**Published:** 2020-07-13

**Authors:** Hamed Seddighi, Farhad Nosrati Nejad, Mehdi Basakha

**Affiliations:** 1 Student Research Committee, University of Social Welfare and Rehabilitation Sciences, Tehran, Iran; 2 Social Welfare Management Department, University of Social Welfare and Rehabilitation Sciences, Tehran, Iran

**Keywords:** Efficiency, Resource allocation, Health care economics and organizations, Health services, Life expectancy

## Abstract

**Background:**

One of the most important issues in public policy and welfare state is health care. Poor management leads to the waste of resources, including money, human resources, facilities, and equipment.

**Aims:**

This paper seeks to answer the question of which eastern Mediterranean countries are more effective in allocating their health resources, and does Iran, in relation to those countries, have an effective health system.

**Methods:**

This study examined technical efficiency among eastern Mediterranean countries in 2018. Data were extracted from Global Health Observatory data World Health Organization. We applied input-oriented Data Envelopment Analysis (DEA) models to estimate efficiency scores. Inputs are Physicians density per 10,000 populations, Total hospital beds per 10,000 populations, Current expenditure on health, % of gross domestic product and outputs are infant survival rate and Life expectancy.

**Results:**

The most efficient health systems in the eastern Mediterranean were Bahrain, Egypt, Iran, Lebanon, Morocco, Oman, Pakistan, Qatar, Tunisia and the United Arab Emirates. The inefficient countries are Iraq, Jordan, Kuwait, Libya, Palestine and Saudi Arabia.

**Conclusions:**

Among the efficient countries, one category of high-entry countries such as Bahrain and Qatar with high input especially in health expenditure had higher output. The second group of countries with lower inputs such as Iran and Morocco has been able to produce similar output with other countries. Also, inefficiency in countries such as Saudi Arabia can be attributed to this with higher input such as health expenditure has lower output such life expectancy and infant survival rate.

## Background

The health sector is one of the most important service sectors and indicators of development [1]. Health systems today form one of the largest sectors of the world's economy [2]. It was found that when GDP increased, health expenditure in general increases [3]. The share of health expenditures in the budgets of low- and middle-income countries is increasing. Countries such as Brazil, Russia, India, China, and South Africa remain to be major drivers of such change since 1990s [4]. Getzen and Jakovljevic confirmed that shifts in GDP growth rates have already reflected heavily on world’s health expenditure landscape [5]. Purchase power parity in terms of health expenditure in low and middle-income countries’ has already grown in some indicators from almost 26.1% in 1995 to 39.7% in 2013, in only a span of 19 years [6]. Over the past few decades, national health spending has risen sharply around the world. It seems that the share of global medical spending by emerging economies is increasing and the share of rich countries is declining [7]. With the rapid growth in the costs of the health sector worldwide, economic experts, managers, and decision-makers are seeking to find new ways to limit costs and increase efficiency [8]. Despite the high volume of resources allocated to the health sector, there is a gap between the growth of the available resources and the resources needed by the health sector, which highlights the need for effective use of resources [9].

The World Health Organization (WHO) in its report emphasized on three goals for the health system [10] including improving health, meeting non-medical needs, and ensuring that financial burden is distributed equitably. In order to achieve this, the WHO has emphasized the performance of health systems [11]. Evaluating the effectiveness and efficiency of health systems is the measurement of the performance of system management. This comparison, when done in a large scale and in the health systems of the countries, shows the results of the selection of managers' policies and practices [10].

Poor management leads to the waste of resources, including money, manpower, buildings, and equipment. Such a loss means that a certain share of services (outputs) can be achieved with fewer resources. By preventing the loss of financial and human resources, they can be used to provide high-quality and cost-effective services [12]. Financial economic analysis provides a logical and specific framework for analyzing important issues in health care [13]. Deciding on the optimal provision of health care is a complex task and requires information about system performance for decision makers. The task of health economists is to analyze issues and report the results of economic assessments in a variety of ways to health policymakers [14]. In addition, with population aging, it is needed national strategies for sustainability of health systems. Countries whose populations are moving faster toward old age need to invest and spend more on health care for the elderly [15]. Non-communicable diseases will continue to be a challenge for low- and middle-income countries. High and out-of-pocket costs will impoverish 150 million people worldwide [16]. Despite significant global health gains, reports show that many low- and middle-income countries are not aligned with global health goals, and the gap between low-income and high-income countries seems unlikely to be narrowed. Current trends show that a significant increase in the resources of the health system requires coordinated action [17]. Poor countries are not able to provide enough funding to meet their health needs in the short to medium term. Governments in these countries have limited ability to collect taxes or health insurance benefits because people are poor and many people work in the informal sector, making it difficult to collect taxes [18].

This paper seeks to answer the question of which countries are more effective in allocating their health resources in Eastern Mediterranean Region, and does Iran, in relation to those countries, have an effective health system?

### Literature

Productivity or efficiency is a criterion for measuring performance, and the value of the input (i.e., what is being used in production) is evaluated by the output (i.e., what is obtained) [19]. Efficiency is a very comprehensive concept, and it is discussed in various areas such as engineering, management, economics, and health. Therefore, different definitions of efficiency are provided in various sources. Farrell defines a firm's efficiency as "to produce an output to a sufficiently large extent than a given input value", and it specifies the technical allocation and economic performance of its types [20]. In many studies were used data envelopment analysis (DEA) method to investigate health systems efficiency [21–24].

It was reviewed 317 studies on health efficiency in a systematic review study, which were divided into two types of micro- and macro-level studies [25]. Micro-level studies evaluated the efficiency and function of health units such as hospitals and clinics [26]. Of course, some recent studies with a macro-level approach have also evaluated the performance of healthcare centers [27–31]. In most of these studies, the outcomes and impacts of the health system are measured in terms of life expectancy, and the main input in most studies is the per capita cost of health. In another study was used DEA method to investigate life expectancy and health expenditure evolution in eastern Europe [23].

It was found in a systematic review of 137 papers that most of reviewed studies employed data envelopment analysis for measuring efficiency in health systems [32]. In an another study was found that Lebanon, Qatar, and Morocco have the most efficient health systems in Middle east and North Africa (Mena) region [33]. Bousmah et al. n their paper analyzed health efficiency in 18 countries between 1995–2012 [34]. In a study about health efficiency in 18 countries between 1995–2012 was found that increasing health expenditure in the MENA region will not result health outcome improvement necessarily and full efficiency of health system [34].

Spinx and Halings [35] evaluated the effects of socioeconomic determinants on the outcome of health care. They used unemployment rate, the level of attribution, and GDP per capita as inputs. Ratzald Roberts et al. [36] provided a comprehensive model of inputs in which social environment, lifestyle, access to health services, and health costs were considered as inputs. Some studies also looked at hospital beds, the number of health care workers and health expenditure as inputs [30, 37]. In a study, two models with different inputs and the same output were studied. In the first model, the number of physicians and beds along with health expenditures were set as input, and in the second model, GDP per capita along with consumption of vegetables and fruits were specified as input. Outputs or outcomes in the health system were considered life expectancy and infant survival rate. By comparing these two models through the data envelopment analysis (DEA) method, the first model was found to be more appropriate [38].

## Methods

In DEA models, the solution for improving inefficient units is to reach the efficiency boundary. The borderline consists of units of efficiency 1. In general, there are two types of solutions for improving inefficient units and reaching the efficiency limit [39]: (A) Decrease inputs without reducing outputs until the unit reaches the border (this approach is referred to as the nature of performance improvement institutes or measures of input-oriented efficiency); (B) increasing outputs by reaching a unit on the efficiency boundary without attracting more inputs (this approach is referred to as the nature of performance improvement or output-oriented performance measurement).

In the DEA models with the input-oriented approach, it is sought to achieve a technical inefficiency ratio that should be reduced in inputs, so that the unit remains within the efficiency boundary without changing the output. However, in the output-driven viewpoint, it is tried to determine the ratio at which outputs should be increased so that the unit can reach the efficiency boundary without changing inputs.

All the Eastern Mediterranean countries base on world health organization, were considered as a decision-maker or decision-making unit (DMU) in the analysis. In term of homogeneity, Six countries, including Afghanistan, Djibouti, Somalia, Sudan, Syria and Yemen, were excluded from the analysis because of low income in compare to other countries [40]. Exclusion criteria is Gross domestic products (GDP), according to the World Bank. In the findings, the results are presented in two ways: BCC and CCR (Charnes, Cooper & Rhodes model), and two input-centered and outbound approach, but for analyzing and discussing the output-oriented approach, because the health system seeks to maximize health rather than keep resources and minimize inputs [37, 38].

**Fig. 1:**
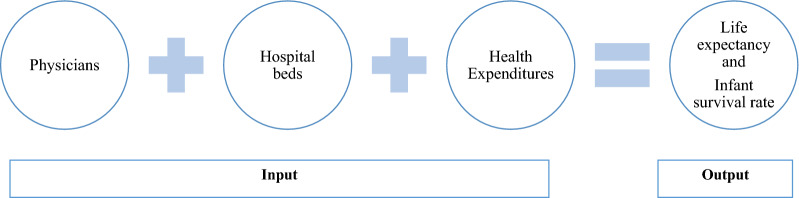
Data envelopment analysis model for healthcare production function

In this study, as shown in Fig. 1, outcomes were considered to be life expectancy at birth and infant survival rate. These two outcomes were chosen based on previous studies, as in many studies they were two of the most commonly used outputs. The reason behind the use of infant survival rate rather than the rate of infant mortality is the nature of DEA which should have a positive outlet. The infant survival rate is obtained by the following formula [41]:

$${\text{ISR}} = 1 - {\text{IMR}}/1000$$

The input and output definitions are provided in Table 1. For analysis, the DEA online solver online application, available at the University of Hagen, Germany, was used. The research data were derived from World Health Organization's Health Surveillance Sect. 2017. All input data can be seen in Table 2.

**Table 1:** Inputs and outputs definition of data envelopment analysis

Source	Parameter	Name	
OECD health data/WHO health observatory	X1	Physicians, density per 1000 population (head counts)	Entrance
OECD health data/WHO health observatory	X2	Total hospital beds, per 1000 population	
OECD health data/WHO health observatory	X3	Current expenditure on health, % of gross domestic product	
OECD health data/WHO health observatory	Y1	Life expectancy	Output
OECD health data/WHO health observatory	Y2	Infant survival rate (calculated from infant mortality rate, deaths per 1 000 live births)

**Table 2:** Data entered into the software

Country	Health expenditure	Physicians	Hospital beds	Infant survival rate	Life expectancy
Bahrain	1243	24.0	18.9	199	76.9
Egypt	178	8.1	14.3	49	73.2
Iran	351	11.4	17.0	75/92,308	75.5
Iraq	292	8.4	13.0	36/03,704	68.9
Jordan	359	14.1	14.0	65/66,667	74.1
Kuwait	1386	29.0	20.4	141/8571	74.7
Lebanon	569	31.0	27.3	141/8571	74.9
Libya	372	19.5	37.0	89/90,909	72.7
Morocco	190	6.3	11.0	40/66,667	74.3
Oman	675	19.6	14.9	99	76.6
Pakistan	36	10.0	6.3	16/85,714	66.4
Palestine	305	21.7	12.8	54/55,556	73.5
Qatar	2106	25.0	12.0	141/8571	78.2
Saudi Arabia	1147	23.9	22.3	75/92,308	74.5
Tunisia	305	13.0	21.8	82/33,333	75.3
United Arab Emirates	1611	22.3	13.6	165/6667	77.1

## Results

Average of life expectancy in the countries studied was 74.17; maximum was for Qatar with 78.2 and minimum was for Pakistan with 66.4, respectively. The average infant survival rate is around 92 in the region, with a maximum of 199 for Bahrain and a minimum of 16.86 for Pakistan. The average health expenditure in the region is $ 695.3, with the highest being $ 2106 and Pakistan's lowest at $ 36. The average number of physicians is 17.9 per ten thousand; this rate is 31 for Lebanon and 6.3 for Morocco. The average of hospital beds in all public and private centers for all patients per 10,000 populations was 17.7.

Based on Table 3 the most efficient health systems in the eastern Mediterranean were Bahrain, Egypt, Iran, Lebanon, Morocco, Oman, Pakistan, Qatar, Tunisia and the United Arab Emirates. The inefficient countries are Iraq, Jordan, Kuwait, Libya, Palestine and Saudi Arabia. If the countries are ranked in terms of efficiency, Jordan and Palestine come in second, followed by Kuwait, Saudi Arabia and Libya, and Iraq fourth. The score of efficiency between countries varies from 1 to 0.91. The efficiency scores are shown in Table 4.

**Table 3:** Health system efficiency ranking

Score	Countries	Rank	Score	Countries	Rank
1	Tunis	1	1	Bahrain	1
1	Emirate	1	1	Egypt	1
0.98	Jordan	2	1	Iran	1
0.98	Palestine	2	1	Lebanon	1
0.96	Kuwait	3	1	Morocco	1
0.96	Saudi Arabia	3	1	Oman	1
0.96	Libya	3	1	Pakistan	1
0.91	Iraq	4	1	Qatar	1

**Table 4:** Health system efficiency in studied countries using the data envelopment analysis method

Inefficient country	Reference countries	Activity level	Inefficiency score
Iraq	Iran	0.329599	0.085384
	Morocco	0.647993	
	Qatar	0.022409	
Jordan	Iran	0.353770	0.015488
	Morocco	0.419751	
	Oman	0.221950	
	Emirates	0.004529	
Kuwait	Oman	0.165197	0.035780
	Qatar	0.323054	
	Emirates	0.511750	
Libya	Bahrain	0.045116	0.036242
	Lebanon	0.093489	
	Tunisia	0.861395	
Palestine	Iran	0.186013	0.019409
	Morocco	0.638622	
	Oman	0.175365	
Saudi Arabia	Oman	0.670161	0.035272
	Qatar	0.329839	

## Discussion

As stated in the introduction, performance enhancement is achieved in two forms [20]. In the first form, inputs are reduced without decreasing outputs until the unit reaches the boundary value (this is the nature of the institutions of performance improvement or performance measurement with the input-oriented nature of the system). In saving resources and reducing input, it must be taken not to reduce the quality of healthcare activities. Rather, the goal is to reduce input if possible while maintaining quality [42]. In the second form, outputs increase until it reaches one unit on the efficiency boundary without absorption of any further inputs (this approach is referred to as the nature of performance improvement performance or output-oriented output efficiency assessment) [20].

This study, according to the type of input and output and the model of health production, two methods can be interpreted. First, the effectiveness of the health system in countries through reduced inputs includes health expenditure, hospital beds and the number of physicians without a decrease in output, that is, infant survival rate and life expectancy at birth. Or an increase in outputs, including the infant survival rate and life expectancy at birth, without increasing health expenditures, the number of physicians and the number of hospital beds will increase efficiency. Considering that the DEA method evaluates each country according to the input and output of several decision-making units of the reference country, the countries that have demonstrated their full effectiveness, according to other countries studied in this research, with fewer or similar inputs had equal outputs. Thus, if in these analysis countries such as Iran and Pakistan achieved complete efficiency, they could be interpreted as having less relative inputs than other countries (e.g., health expenditures, hospital beds, and the number of physicians) and almost the same output in terms of healthy life expectancy and infant survival rate compared to the other countries. Obrizan et al. (2018) in a study on health expenditures and longevity indicated that that countries' health expenditure has inconsistent effects on life expectancy. Furthermore, the effects are larger for countries at the left margin of the longevity distribution, and higher health costs can have significant returns in countries with low-longevity [43].

Countries that are more inefficient like Kuwait and Saudi Arabia can be attributed to the have a much larger influx than other countries, which means either much more health expenditure per capita or per capita physician and hospital beds, or combining these three high rates, while in outputs, there was not much difference in terms of healthy life expectancy at birth and infant survival rate. For example, life expectancy in the Saudi Arabia was the same as that in Iran (74.5 years compared to 75 years), while its health expenditure per capita was more than three times as much as Iran, and thus, its health system has been shown to be inefficient in DEA compared to Iran. It was indicated that lifestyle, health behavioral health beliefs and shared culture are effective on healthcare system quality besides of the physicians' number and hospital beds [44]. Arab countries, more than other countries, spend public resources on education and health, but they do not have the appropriate output to investigate this inefficiency [45]. However, health status were influenced by health care expenditure through improving life expectancy at birth, reducing death and infant mortality rates in low-income countries such as countries in sub-Saharan Africa region [46]. Healthy life expectancy has more than doubled in the past 200 years, but in the past few decades its growth is not as high with the increase in life expectancy reaching over 70 years globally. This growth is much slower, especially in middle income and higher income countries, with an average of 81.4 years. Therefore, the growth of life expectancy at birth is not expected to be proportionate to the growth of health expenditure, hospital beds, physicians, and other health system inputs. Compared to the past, people have a longer and healthier life in the present age, but this achievement has cost a lot. Advances in treatment, as well as the introduction of new drugs, have also increased longevity [47]. Demand in the healthcare sector has increased significantly due to the spread of non-communicable diseases, home care, and the high cost of treatment and care in the last years of life. However, technological innovations in medicine have not yet expanded sufficiently in terms of cost-effectiveness resource allocation [48]. Universal Health Coverage (UHC) is a comprehensive health system approach that facilitates a wide range of health services and significantly improves the life expectancy at birth and healthy life expectancy [49]. In addition, it is indicating that public health programs like sanitation and vaccination also affect life expectancy [49]. Even with rising health spending, low and middle-income countries need to coordinate between promoting public health, controlling non-communicable diseases, and improving population health, but this is currently a major challenge [50].

## Conclusion

In this study, we evaluated the efficiency of health systems in eastern Mediterranean countries using the DEA method. The health system of Iran compared to the East Mediterranean countries was fully effective, indicating that in terms of life expectancy and infant survival rate (two very important health system indicators), Iran compared in proportion to its inputs (i.e., health expenditures, physicians' ratio, and hospital beds) is fairly well-suited. The efficiency of Iran's health system is mostly related to inputs than health outcomes. Also, this study showed increasing health expenditure and healthcare facilities will not guarantee better performance in healthcare. In some countries with weak output in life expectancy and infant survival rate, increasing output would be effective. However, in eastern Mediterranean countries, balance between inputs and outputs should consider for better allocating resources.

## Data Availability

The data supporting the findings in this study is publicly available at: https://apps.who.int/gho/data/node.main
